# Correction: Tuning the spontaneous emission of CdTe quantum dots with hybrid silicon–gold nanogaps

**DOI:** 10.1039/d5ra90101d

**Published:** 2025-09-08

**Authors:** Areeg Al-hamadani, Ali Al-Dulaimi, Theresa Bartschmid, Johannes Menath, Alina Muravitskaya, Nicolas Vogel, Gilles R. Bourret, Jean-Sebastien G. Bouillard, Ali M. Adawi

**Affiliations:** a Department of Physics, University of Hull Cottingham Road UK a.adawi@hull.ac.uk j.bouillard@hull.ac.uk; b Department of Physics, College of Science, University of Diyala Diyala Governorate Baquba City 32001 Iraq; c Department of Chemistry and Physics of Materials, University of Salzburg A-5020 Salzburg Austria; d Friedrich-Alexander-Universität Erlangen-Nürnberg (FAU), Institute of Interfaces and Particle Technology 91058 Erlangen Germany; e Department of Physics, School of Science, The University of Jordan Amman 11942 Jordan

## Abstract

Correction for ‘Tuning the spontaneous emission of CdTe quantum dots with hybrid silicon–gold nanogaps’ by Areeg Al-hamadani *et al.*, *RSC Adv.*, 2025, **15**, 29053–29062, https://doi.org/10.1039/D5RA04583E.

The authors regret an error in the spelling of Ali Al-Dulaimi's name in the original manuscript. The correct spelling is as shown in this Correction article.

The Royal Society of Chemistry apologises for these errors and any consequent inconvenience to authors and readers.

